# Virtual reality in simulation-based emergency skills training: A systematic review with a narrative synthesis

**DOI:** 10.1016/j.resplu.2023.100484

**Published:** 2023-10-21

**Authors:** Jonathan R. Abbas, Michael M.H. Chu, Ceyon Jeyarajah, Rachel Isba, Antony Payton, Brendan McGrath, Neil Tolley, Iain Bruce

**Affiliations:** a The University of Manchester, Oxford Road, Manchester M13 9PL, United Kingdom; b Manchester University NHS Foundation Trust, Oxford Road, Manchester M13 9WL, United Kingdom; c Lancaster Medical School, Lancaster University, Lancaster LA1 4YW, United Kingdom; d VREvo Ltd, The University of Manchester Core Technology Facility, 46 Grafton Street, Manchester, M13 9NT; e Manchester Academic Critical Care, Division of Infection, Immunity and Respiratory Medicine, School of Biological Sciences, Faculty of Biology Medicine and Health, University of Manchester, Manchester Academic Health Sciences Centre, Manchester, United Kingdom; f Imperial College Healthcare NHS Trust, The Bays, South Wharf Road, St Mary's Hospital, London W2 1NY, United Kingdom; g Alder Hey Children's NHS Foundation Trust, Eaton Road Liverpool, L12 2AP, United Kingdom

**Keywords:** Virtual Reality, Systematic Review, Simulation, Emergency Simulation

## Abstract

**Objective:**

An important role is predicted for virtual reality (VR) in the future of medical education. We performed a systematic review of the literature with a narrative synthesis, to examine the current evidence for VR in simulation-based emergency skills training. We broadly define emergency skills as any clinical skill used in the emergency care of patients across all clinical settings.

**Methods:**

This systematic review followed the Preferred Reporting Items for Systematic Reviews and Meta Analyses (PRISMA) guidelines. The data sources accessed during this study included: PubMed, CINAHL, EMBASE, AMED, EMCARE, HMIC, BNI, PsychINFO, Medline, CENTRAL, SCOPUS, Web of Science, BIOSIS Citation Index, ERIC, ACM Digital Library, IEEE Xplore, and ProQuest Dissertations and Thesis Global. Cochrane’s Rob 2 and ROBVIS tools were used during study quality assessment. No ethical review was required for this work.

**Results:**

Thirty-four articles published between 14th March 1998 and 1st March 2022 were included in this review. Studies were predominantly conducted in the USA and Europe and focussed on a variety of healthcare disciplines including medical, nursing, and allied health. VR education was delivered using head-mounted displays, Cave Automatic Virtual Environment systems, and bespoke setups. These systems delivered education in a variety of areas (emergency medicine, equipment training, obstetrics, and basic/advanced life support). Subjective potential advantages of this technology included realism, replayability, and time-effectiveness. Reports of adverse events were low in frequency across the included studies. Whilst clear educational benefit was generally noted, this was not reflected in changes to patient-based outcomes.

**Conclusion:**

There may be educational benefit to using VR in the context of simulation-based emergency skills training including knowledge gain and retention, skill performance, acceptability, usability, and validity. Currently, there is insufficient evidence to demonstrate clear cost-effectiveness, or direct improvement of patient or institutional outcomes, at this stage.

## Introduction

Clinical reasoning, decision-making, and psychomotor skills form the cornerstones of the delivery of safe and effective emergency medical care.^1^, ^2^, ^3^ Clinical skill development is a complex interplay between theoretical knowledge, cognitive ability, and motor skill development.^4^ Further pedagogical consideration must be made around the influence of non-technical skills and cognitive bias which have implications in reducing harm, improving patient outcomes, and reducing the litigation burden on healthcare organisations around the world.^5^, ^6^, ^7^

Traditional high-fidelity simulation is considered the gold-standard of emergency skills training, and in the last decade, the number of specialist centres is growing significantly.^8^, ^9^ Traditional simulation training occurs in a variety of settings, utilising a wide range of techniques, which can include the use of physical mannequins, part-task trainers, table-top exercises, cadavers, role-play, or in-situ techniques.^10^ Simulation has been demonstrated to address the theory–practice gap across different healthcare professionals.^11^ Simulation activities predominantly take place within the acute-care setting, educational institution, or purpose-built simulation suite.^12^ Disadvantages of traditional simulation include: high-cost, large space requirement, facilitator expertise requirement, ethical issues in human or animal tissue use, and difficulty in, or high-cost of, repeating the educational experience.^13^, ^14^, ^15^, ^16^

Virtual reality (VR) can be defined as “a three-dimensional (3D) computer-generated simulated space, which attempts to replicate real world or imaginary environments and interactions, thereby supporting work, education, recreation, and health”.^17^ It exists as a sub-type of extended reality technology (XRT) alongside two other main technological sub-groups: augmented reality (AR) and mixed reality (MR). Augmented reality (AR) is the overlaying of computer generated images onto a real-life viewing window.^17^ This has been popularised in recent times through mobile gaming and social media.^12^ MR can be viewed as a stepwise technological development, building on AR technology whereby the computer-generated content is anchored to a reference point within in the 3D real-world view.^18^ The spatial anchoring of digital assets enables the user to manipulate these objects within the 3D space.^18^

Throughout this article the use of the term 'emergency skills training’ can be defined as any clinical skill which is used to deliver emergency care to patients. For added clarity, as long as the skill is in the context of emergency patient care, it may be inclusive of technical and non-technical skills, clinical reasoning, teamwork and communication. In this systematic review we aimed to identify, evaluate, and summarise the literature around emergency VR simulation training in order to highlight areas in greatest need of further work (research methodology, hardware and software selection, usability, comfort, or side effects). Thorough literature reviews of XRT as a whole, and reviews focused on specific emergency skills such as open trauma surgery training, disaster medicine, or airway skills training have been written.^19^, ^20^, ^21^, ^22^ To date, there has been no attempt to systematically review the literature around VR and simulation-based emergency skills training.

## Methods

### Search strategy and data selection

This systematic literature review followed the Preferred Reporting Items for Systematic Reviews and Meta Analyses (PRISMA) guidelines.^23^ The study protocol was published in advance of data collection (PROSPERO ID: CRD42020205636).^24^ We consulted specialist academic librarians to support the formulation of the following search strategy which was used to search all article data fields^25^:

(doctor* OR physician* OR nurs* OR medic* OR student OR surgeon* OR health*) AND (“virtual reality” OR “augmented reality” OR “mixed reality” OR immers* OR “computer-assisted instruction”) AND (“simulation training” OR “high fidelity simulation training” OR “patient simulation” OR simulation OR training OR “medical training” OR “medical education”) AND (emergency OR acute OR urgent OR “emergency care” OR “emergency treatment” OR “emergency medicine” OR “emergency therapy”)

In an attempt to ensure that relevant articles were not overlooked, given the known complexities of XRT terminology, we decided to include the terms “Augmented Reality” and “Mixed Reality”.^17^ The individual databases accessed using the above search terms were as follows: PubMed, CINAHL, EMBASE, AMED, EMCARE, HMIC, BNI, PsychINFO, Medline, CENTRAL, SCOPUS, Web of Science, BIOSIS Citation Index, ERIC, ACM Digital Library, IEEE Xplore, and ProQuest Dissertations and Thesis Global. The search period was between the years 1998 and 2022. The final database searches were conducted on the 16th August 2022.

A detailed list of inclusion and exclusion criteria can be found in Table 1. In summary, we aimed to include articles which investigated the efficacy of VR simulation-based emergency skills training within a population of nurses, doctors, and allied health professionals of any seniority including their students. The PICO model criteria related to this study are listed below and described in more detail in Table 1:

**P (population)** – doctors, nurses, or allied health professionals and healthcare students.
**I (intervention)** – specific examples of any deployment of VR technology which has been designed to teach any simulation-based emergency skill.
**C (comparator)** – any alternative forms of training the same skill, or no training given.
**(outcome)** – clinical competence, or surrogate markers of clinical competence. These may have been measured by the reporting of patient outcomes, skill performance in real or simulated environments, or knowledge gain. Additional outcomes of interest include participant motivation, stress levels, usability, acceptability, perceived competence and cost.

**Table 1:** A table displaying a comprehensive list of inclusion and exclusion criteria.

Inclusion	Exclusion
Published in English	Not published in English
Original research paper	Non-research article Letters to the editor of journalsEditorials or opinion piecesNon-systematic scoping literature reviews
Peer-reviewed	Not peer-reviewed
Finished work	Work in progress
Full-text available online or through library or inter-library loan	Full text report unavailable
Study population to include doctor, nurse or allied health professional at any level of seniority, inclusive of any level of pre-clinical students of each discipline. Allied health professionals may include; physiotherapist, dentist, speech and language therapist etc.	Non-healthcare worker, i.e. fire crew or police.
Intervention - VR training of the above population within the field of emergency medicine or surgery. Examples of emergency training applications may include (not exhaustive): • Emergency medicine • Emergency surgery • Emergency procedure • Disaster/major incident • Warzone medicine	VR interventions not related to training including therapeutic applications.
Intervention meets criteria for VR as defined by Abbas et al.^16^: • Computer based – portable or tethered • Immersive • 1st person • Hardware can be head mounted	Non-VR intervention
Comparison – we can include papers which compare intervention to: 1. Traditional methodology 2. Electronic methodology 3. Other technology enhanced interventions	
Outcome – Outcomes assessing the efficacy of the educational tool to include subjective and objective outcome measures and user experience testing. 1. Knowledge transfer 2. Skill performance 3. Patient adverse events/complications	No measured outcome which investigates the efficacy of the educational tool

We acknowledge the minor deviation from the PROSPERO protocol to include non-validated outcome measures of performance and the inclusion of measures of participant satisfaction, motivation, and acceptability. This decision was made due to the inherent value of reporting these literature data points in the context of novel technology enhanced learning.

### Study selection and screening process

Following search completion and de-duplication using Endnote citation manager, the titles and abstracts of the articles were screened by two independent reviewers (JA and MC). Each reviewer selected articles from this screening process to read the full-texts, and decide upon inclusion based on the criteria outlined in Table 1. This screening process was administered blindly, with complete independence, and was facilitated by the use of Rayyan Intelligent Systematic Review software.^26^ Once the reviewers had made their independent decisions about inclusion and exclusion of each article, the process was unblinded and conflicts were resolved by consensus discussion. Where consensus could not be agreed, a third reviewer (CJ) was consulted to make the final decision.

### Data extraction and quality assessment

Utilising the Google Forms cloud-based database software, a data extraction template was constructed based on the Cochrane Handbook for Systematic Reviews of Interventions.^25^ Our data extraction tool was piloted on five papers in order to refine the process, prior to finalisation and wider use. For randomised controlled trials (RCTs) the RoB 2 tool combined with the ROBVIS visualisation tool were used in the assessment of bias.^27^ This assessment was performed by two independent reviewers and the scores aggregated (JA and CJ). Quality assessment of non-RCTs was assessed following adaptation of the tools presented by the National Heart, Lung, and Blood Institute.^28^

### Data reporting and analysis

A narrative synthesis of the included articles was performed focussing on the following key areas:

(1) Study characteristics
(2) Description of the VR interventions
(3) Assessment of evidence quality
(4) Outcomes measured throughout the literature
(5) Adverse effects including stress and side-effects
(6) Cost analysis

## Results

### Study characteristics

**Fig. 1:**
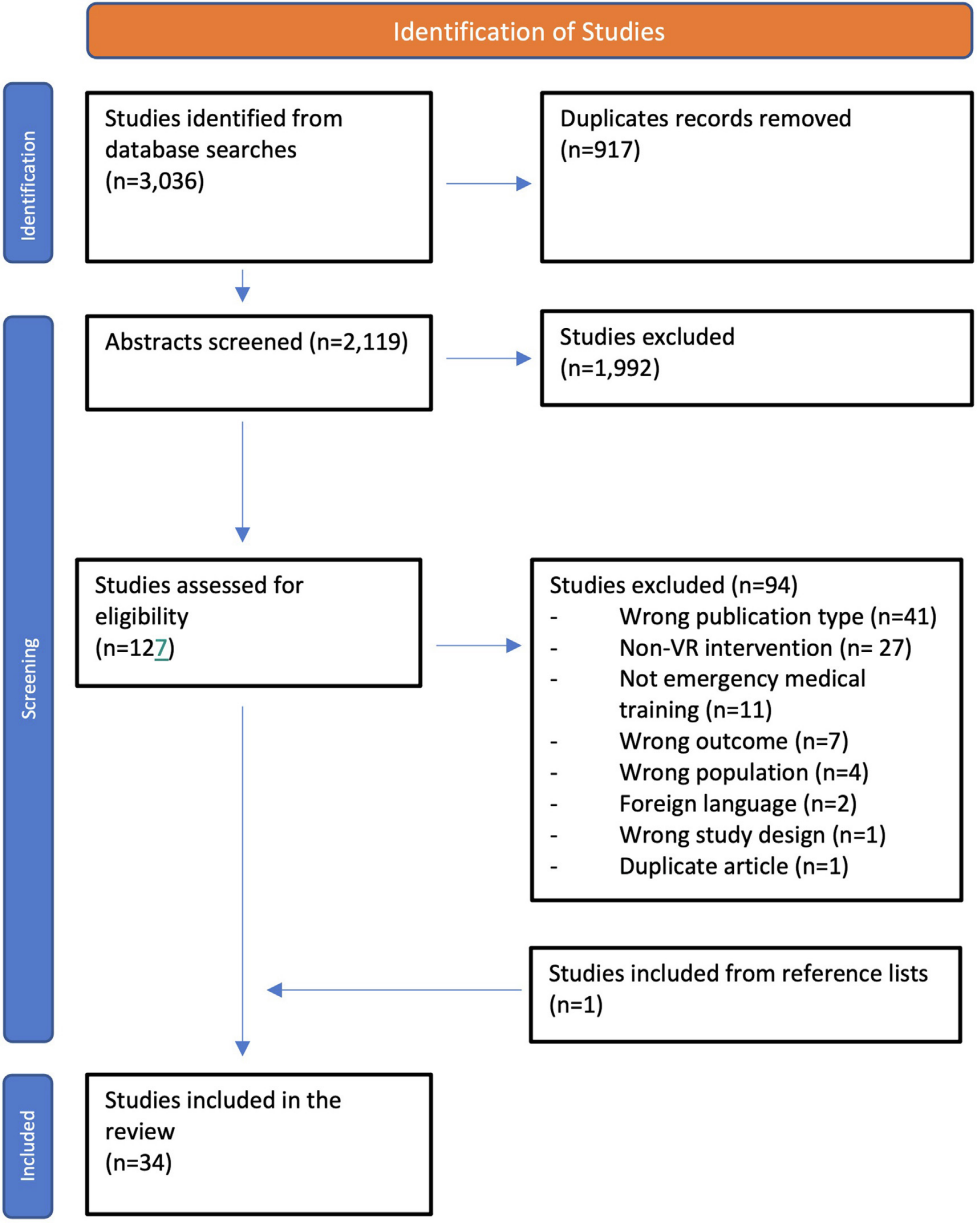
Search results summarised in a PRISMA style flowchart as described by Moher et al.^74^

Initial searches revealed 3,036 potential articles for inclusion. Following de-duplication 2,119 abstracts were screened, 125 articles underwent full-text review, of which 33 studies were selected for inclusion. A single study was added following review of the reference lists. A total of 34 articles published between 14th March 1998 and 1st March 2022 were included in the analysis. A full screening process is presented in the PRISMA flowchart in Fig. 1. Studies were conducted in the following countries: United States of America (USA) (*n* = 20), Australia (*n* = 2) and Italy (*n* = 2), with a single further study published in each of Colombia, Spain, Norway, Turkey, France, Singapore, Indonesia, Ireland, United Kingdom (UK), and Germany. The key characteristics including authorship, publication dates, methodology and results are summarised in Supplementary Material 1.

### Description of intervention

The VR interventions studied in the included articles utilised a variety of hardware and software interfaces. Twenty-two used VR head mounted displays (HMD),^29^, ^30^, ^31^, ^32^, ^33^, ^34^, ^35^, ^36^, ^37^, ^38^, ^39^, ^40^, ^41^, ^42^, ^43^, ^44^, ^45^, ^46^, ^47^, ^48^, ^49^, ^50^ four studies utilised Cave Automated Virtual Environment (CAVE) VR systems,^51^, ^52^, ^53^, ^54^ five utilised bespoke proprietary VR setups^55^, ^56^, ^57^, ^58^, ^59^ and in two papers the hardware was not clearly specified.^60^, ^61^ Educational tools were designed to teach a variety of skills in the following medical speciality domains: paediatric/adult medicine,^29^, ^31^, ^37^, ^38^, ^40^, ^45^, ^47^, ^62^, ^61^ cardiology,^30^ anaesthetics including airway skills,^55^, ^57^, ^59^, ^60^ disaster/mass casualty training,^32^, ^34^, ^35^, ^39^, ^45^, ^49^, ^51^, ^53^, ^54^, ^56^ operating room fire/evacuation training,^36^, ^43^, ^56^, ^58^ emergency equipment/vehicle orientation,^33^, ^58^ obstetric emergency,^44^ and basic/advanced life support training.^41^, ^42^, ^46^, ^48^, ^50^, ^52^

### Assessment of evidence quality

**Fig. 2:**
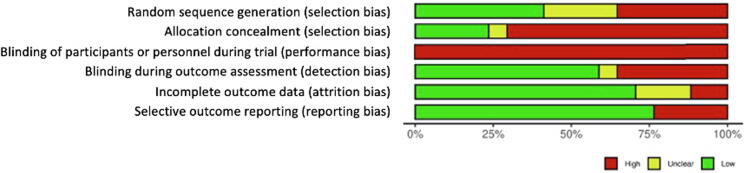
Risk of bias graphical representation. Represented as percentages displayed in this figure is the combined authors judgements for risk of bias across all included studies.

**Fig. 3:**
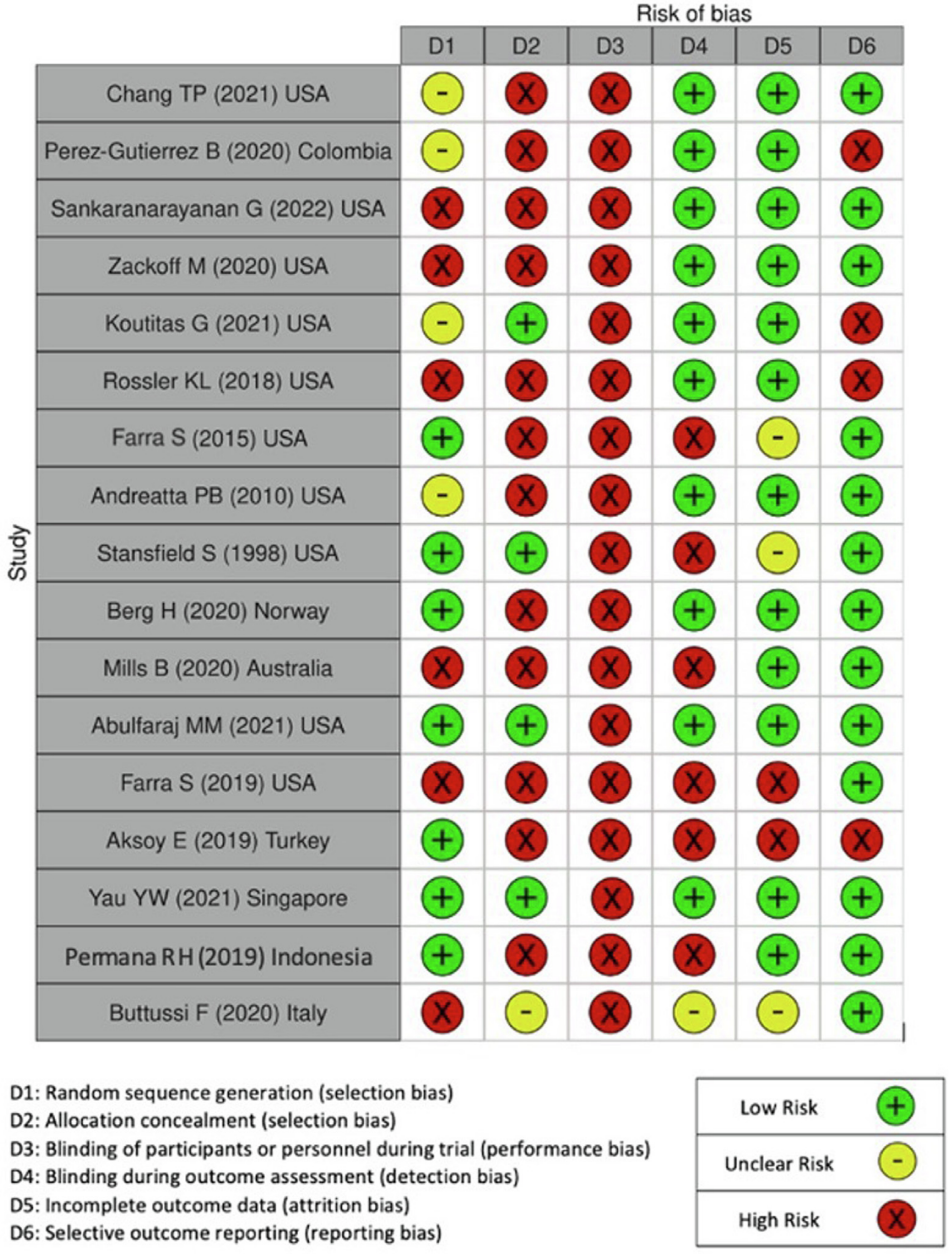
Risk of bias graphical representation. Represented individual domain risk of bias for each study.

Of the included studies, 17 implemented an RCT design, 14 studies were observational in nature, and a further three were cohort studies. Of the RCTs, 15 had two arms, and two studies had three experimental arms each. Control arms in these studies varied including mannequin simulation (*n* = 13), deployments of the same VR hardware, but with either differences in the software packages or differences in exact timing of intervention exposure (*n* = 3), or no training (*n* = 1). The bias assessments are presented in Fig. 2, Fig. 3. Overall four trials were considered to have low risk of bias,^31^, ^39^, ^40^, ^60^ with the remaining 13 considered to have considerable risk due to lack of clearly described random sequence generation,^29^, ^30^, ^33^, ^35^, ^38^, ^42^, ^51^, ^56^, ^58^, ^59^ projects not blinding outcome assessors,^35^, ^41^, ^56^, ^61^, ^62^ and evidence of incomplete or selective reporting.^30^, ^33^, ^41^, ^58^, ^62^ Participant blinding was not possible, however in 10 studies the outcome assessors were blinded.

There was little concordance in terms of interventions, research methodology, outcomes, and the outcome measure instruments. This factor, combined with missing data, and differences in data presentation meant that pooling of results and therefore a meta-analysis was not possible.^25^

When assessing the quality of the VR interventions included in this study, it was important to think about how the various outcomes may impact an educator or institution in terms of decision to purchase, and therefore use VR. The interventions described throughout the review were assessed with a wide variety of outcomes, and the importance of these will subjectively differ depending on which institution, educator or learner is considering the educational value.

### Knowledge

A measurement of knowledge was taken in nine of the included articles.^41^, ^42^, ^45^, ^50^, ^51^, ^56^, ^58^, ^61^, ^62^ In all studies, knowledge was measured using a pre- and post-intervention written assessment model. Statistical validity of knowledge testing tools was variable. Farra et al. utilised an adapted version of the Federal Emergency Management Agency (FEMA)^63^ multiple-choice questionnaire (MCQ) which is utilised in the official course documentation at FEMA.^56^ Harrington et al. utilised the widely-accepted Advanced Trauma Life Support MCQ.^50^ The remainder of the articles reporting knowledge did so with un-validated knowledge test scores.

VR interventions lead to an increased gain in knowledge in four studies.^41^, ^51^, ^58^, ^61^ Furthermore, Permana et al. demonstrated highest knowledge gain when VR was utilised alone in the training of nurses in several clinical areas, when compared to VR combined with didactic education.^61^ Two articles noticed significant increases in knowledge following VR education, however there was no difference between VR and the control groups.^56^, ^62^ Lerner et al. found, when using VR hardware to teach management of paediatric anaphylaxis in emergency physicians, that there was no increase in knowledge.^45^

Statement of synthesis for VR and knowledge transfer – VR is likely to lead to a positive effect on the knowledge of the learners, as compared with traditional educational tools or in the absence of a comparator.

### Performance

The majority of the included articles reported performance as an educational outcome measure, however, 13 studies did not.^30^, ^34^, ^37^, ^39^, ^41^, ^43^, ^44^, ^45^, ^46^, ^47^, ^48^, ^57^, ^61^ Included in these, were two studies where skill performance-based outcome data was collected, but not reported in the article.^44^, ^45^ The study by Rossler KL et al. was conducted during a routine fire safety course and performance was simply measured by whether the candidates passed, or failed the course.^58^ As all candidates passed, it can be deduced that there was no difference between VR, or traditional didactive lecture-based training.^58^ The specific methodology for measuring performance varied significantly throughout the studies. The predominant process of measuring performance was with subjective and objective tools, in a post-intervention assessment. This was done with the participant undertaking an activity in a VR experience,^29^, ^36^, ^49^, ^55^ a task trainer,^59^ during a real life activity,^33^ during a non-VR simulation,^31^, ^32^, ^40^, ^42^, ^50^, ^51^, ^52^, ^53^, ^56^, ^60^, ^62^ or during a traditional Objective Structured Clinical Examination style assessment.^38^, ^58^ Alternatively, performance was measured during delivery of the intervention, and therefore assessment was different depending on which arm of the study the participant was assigned 35, 54. Interestingly, none of the studies included in this systematic review assessed whether the VR interventions had an effect on patient outcomes. Within the studies noted to collect participant performance metrics, the outcome measure instruments (OMIs) used were also highly variable. OMI’s included time to task completion or key specific interventions, observed simulation score, accuracy of findings, measure of participants’ practical understanding of the simulation, overall task success, economy of movement measures, individual component success, simulated skill negative effect rate (such as simulated death), overall course pass rate, and the use of a validated psychomotor skill score.

Regarding skill performance, a variety of conclusions were drawn. Berg et al. demonstrated, in a cohort of medical and nursing students, non-inferiority between VR and self-directed mannequin training for the ‘ABCDE’ approach to the assessment of the acutely unwell patient.^31^ Four studies reported performance metrics in order to demonstrate content and construct validity rather than measuring educational effectiveness against a control.^29^, ^36^, ^49^, ^50^ Vincent et al. and Qi et al. show that VR improved skill performance with subsequent simulation trials.^36^, ^49^ By finding that senior physicians performed better than juniors, construct validity of the interventions were found in two studies.^29^, ^50^

In general, the comparative studies showed that skill performance improved significantly. Only two studies found that control groups outperformed VR educational interventions.^52^, ^54^ Wier et al. noted better performance in simulation (measured by time to key specific interventions and competence scores) in the control group through team based emergency trauma training, compared the intervention group which utilised CAVE technology.^54^ Rushton et al. demonstrated improved performance in standard Basic Life Support (BLS) trained individuals than VR trained, where performance in simulation was measured by overall BLS scores, chest compression quality, and ventilation scores.^52^ Two studies noted improved skill performance when also training BLS skills, however these articles were observational in nature.^46^, ^48^ Additionally, Buttussi et al. noted mixed results when teaching BLS training in VR.^42^ Other studies noted a mixed effect on performance with no significant differences found in two trials,^32^, ^40^ and mixed performance scores depending on exact OMI component analysed.^53^

Statement of synthesis for VR and skill performance - The included data suggest that VR may improve performance of a skill in a simulated or virtual environment as compared with traditional educational tools or in the absence of a comparator.

### Usability and participant satisfaction

A total of 17 articles reported either usability,^30^, ^31^, ^39^, ^40^, ^45^, ^48^, ^50^, ^53^, ^57^ acceptability,^37^, ^39^ or participant satisfaction.^34^, ^35^, ^36^, ^46^, ^47^, ^49^, ^50^, ^52^ In all three domains there were a variety of OMIs used to measure the above.

Usability was most frequently measured using the validated System Usability Scale.^30^, ^31^, ^40^, ^45^, ^64^ This is a reliable, time efficient, 10-item usability scale in the form of a Likert questionnaire.^64^ It focusses on a user’s perceived confidence in the system, opinion around a system’s consistency, and whether they feel others will find it easy to use.^64^ Other methods of assessing usability include validated questionnaires such as the Simulation Design Scale; author-designed un-validated questionnaires, and qualitative interviews.^65^ Usability scores were high across all VR interventions. Although high levels of usability were noted in the cardiopulmonary resuscitation training VR experience presented by Semeraro et al., 8/39 participants reported difficulty getting to the simulated patient.^48^ Whilst Qi et al. found very high levels of usability in their VR surgical cricothyroidotomy intervention, the lowest ratings were in relation to instrument handling.^57^ Interestingly, Berg et al. found that participants favoured VR over mannequin simulation for learning the ‘ABCDE’ assessment.^31^

Participant satisfaction across all VR experiences was high. Several authors noted high levels of engagement,^34^ enjoyment,^34^, ^46^, ^47^, ^50^ realism.^36^, ^47^, ^50^, ^66^ There was a suggestion that VR was potentially more immersive, and even preferable than traditional methods for teaching paediatric mass triage, and operating room fire safety.^34^, ^36^ Mills et al. noted that satisfaction was equivalent between in-situ simulation and VR groups.^35^ These users felt that realism and fidelity was higher in VR, suggesting therefore that VR could be a potential alternative to high-fidelity simulation.^35^ Ralston et al. described the evaluation of a HMD delivered VR paediatric critical care simulation where senior educators gave unanimously positive feedback.^47^

Statement of synthesis for VR usability, acceptability and participant satisfaction – the data strongly suggests that VR is usable, satisfactory, and in some cases favoured over traditional educational tools.

### Stress

Three studies measured heart rate^29^, ^35^ or salivary cortisol levels^29^, ^32^ as a surrogate markers used to measure the level of participant stress. None reported any abnormalities in these physiological markers. Mills et al. reported higher stress levels in participants undergoing traditional live in-situ triage training than in VR.^35^ Price et al. noted equivalent rise salivary cortisol levels after the VR and traditional in-situ educational sessions.^32^ Chang et al. demonstrated that stress levels measured by the participant’s heart rate and salivary cortisol levels were lower in senior clinicians who were undertaking the VR experiences when compared to the junior doctors.^29^

Statement of synthesis for VR and participant stress – there is insufficient data to draw strong conclusions.

### Adverse effects

Adverse effects were specifically described in six articles which included motion sickness and dizziness. Table 2 outlines which articles describe adverse effects of the VR intervention, the frequency with which they were experienced, and the hardware used.

**Table 2:** A summary of adverse effects specifically discussed detailing the type of hardware, adverse effect frequency and exact symptoms experienced.

Citation *country*	Technology	Numbers experienced (% of cohort)	Symptom detail
Wilkerson W (2008) *USA*	Cave automated virtual environment	Not detailed	Motion sickness
Abulfaraj MM (2021) *USA*	VR HMD (Oculus Rift)	1 (2.4%)	Dizziness
Aksoy E (2019) *Turkey*	VR HMD (Unknown brand)	Not detailed	Motion sickness
Lerner D (2020) *Germany*	VR HMD (HTC Vive)	2 (11.1%)	Motion sickness
Rushton MA (2020) *UK*	Cave automated virtual environment	4 (1.9%)	Dizziness
Harrington CM (2018) *Ireland*	VR HMD (Samsung Gear)	Not detailed but Likert score 2.0/7 median	Motion sickness

Statement of synthesis for VR and adverse effects – VR may be used with little risk of adverse effects.

### Cost

Of the 34 included articles, three specifically detailed the cost of developing or running the VR interventions, which is summarised in Table 3.^34^, ^35^, ^51^ Andreatta et al. and Lowe et al. describe the cost of developing their VR interventions.^34^, ^51^ Predictably Andreatta et al. describe higher development costs between $20,000 and $100,000 USD per VR case as this was focused around fully-immersive VR simulation.^51^ Conversely, in the study by Lowe et al. predominantly 360 degree video was used which attracted a lower development cost of only $7,000 USD.^34^ Neither study discussed other costs involved in delivering VR training including staff time, space requirements, hardware costs and the maintenance requirements. A fuller cost-analysis was performed by Mills et al. indicating that VR training delivery in the context of mass casualty education was less than 10% of the cost of traditional in-situ training ($712.04 USD for a half-day VR training course, compared with a half-day in-situ simulation training course $9,413.71).^35^ However, they did not account for pre-existing infrastructure in the cost-analysis, as recommended by Maloney et al.^35^, ^67^ Despite only three articles detailing the cost of the VR interventions, authors throughout the included articles describe VR as being cost effective.

**Table 3:** A summary of specific cost analysis data extracted from the included studies.

Title and author	Nature of cost analysis	Results
Andreatta PB et al. Virtual reality triage training provides a viable solution for disaster-preparedness^51^	Specific total cost of developing VR intervention (No description of running, maintenance, or hardware costs included)	$20,000–$100,000 per VR scenario
Lowe J et al. 360 virtual reality paediatric mass casualty incident: A cross sectional observational study of triage and out-of-hospital intervention accuracy at a national conference^34^	Specific total cost of developing VR intervention(No description of running, maintenance, or hardware costs included)	$7,000 to produce the entire library of VR training content
Mills B et al. Virtual Reality Triage Training Can Provide Comparable Simulation Efficacy For Paramedicine Students Compared To Live Simulation-Based Scenarios^35^	Full cost analysis which excludes development costs, or the cost of pre-existing infrastructure	$712.04 USD for half day VR intervention delivery $9,413.71 for a half-day in-situ simulation trainingCost neutrality at 145 participants

Statement of synthesis for VR and cost – although authors indicate cost-effectiveness, there is insufficient evidence to draw this conclusion.

## Discussion

It is clear from this review that widespread, significant, and sustained interest in VR technology as applied to emergency medical education exists. Whilst considering evaluating VR in simulation, it is useful to highlight some of the similarities and differences between VR training and traditional mannequin-based simulation:

**1. Immersion and realism** – whilst mannequin simulation, especially in high-fidelity multi-disciplinary exercises can be immersive, VR training has the ability to add dynamic and engaging 3D environments to the experience.^12^

**2. Versatility and adaptability** – mannequin simulation limits the educators to a physical, pre-defined model (unless utilising advanced 3D printing techniques).^68^ In VR there is limitless capability to change, update, and add features to a training experience (at cost, time, and effort equivalent to the proposed change).^69^

**3. Cost** – this review has demonstrated the cost of VR is not well defined, but considerations around hardware, maintenance, storage, training, and technological development costs of VR apply. The cost of traditional mannequin simulation is variable and has been identified as a potential barrier to adoption.^16^

**4. Accessibility –** VR is becoming more comfortable and accessible over time, however all forms of simulation-based emergency skills training (including VR solutions) may be criticised for exclusion of learners with certain disabilities. Additionally, important design considerations exist when making decisions around implementation of haptic feedback devices which require varying levels of manual dexterity. This is not dissimilar to physical props used in mannequin based simulation training, tabletop exercises, or cadaveric dissection. This is an area in need of academic focus, due to paucity of literature available.

The included trials represent a broad range of educational content, across many disciplines. We can deduce that across the breadth of learning outcomes a high level of usability, enjoyment and overall acceptability exists. A common theme throughout this systematic review is that VR education targets rare event training. Several factors contribute to the challenge of simulating rare emergency events with high-fidelity.^70^ These included the high costs of live acting, technologically advanced mannequins, and high-quality make-up art all of which are required to be live, in-situ.^70^ It is noted that the literature strongly supports mannequin-based simulation in rare event training, but these activities need repeating for knowledge and skill retention to exist beyond 2–6 months.^71^

Any healthcare educational institution is tasked with producing clinically competent individuals who require a complex combination of knowledge, clinical reasoning skills, communication skills and psychomotor ability.^72^, ^73^ Teaching these individual components which together, form clinical competence, is a complex task both in terms of educational design, but also in terms of validation through research.

In terms of researcher time and project expense, measuring theoretical knowledge using written tests is simpler than other forms of assessment of competence. In the context of emergency VR education, this review has revealed that VR is highly likely to increase participant knowledge. Mixed results were however seen when VR was directly compared to traditional simulation-based education. VR, being a highly interactive audio-visual learning tool, may lend itself better to transferring experiential learning rather than raw, written knowledge as highlighted by Larson et al. in the context of laparoscopic surgical training.^74^

Overall clinical skill is often measured by direct observation from senior staff with semi-subjective judgement.^74^ Assessment by observation on real patients, especially in rare events such as operating room fires and mass casualty incidents, requires far greater research resource than assessments in simulation.^75^, ^76^ Additionally, measuring hard end points such as mortality and morbidity are simply impractical in medical education.^74^ None of the included papers assess performance on real patients, however Koutitas et al. described clinical skill competence following VR training as applied to knowledge of a real ambulance bus.^33^

Articles predominantly report assessment during VR experiences, task trainers, and simulations which if the results are positive for clinical skill gain represent competence of a given clinical skill. The strength of the body of presented evidence lies in this domain, and we can deduce that it is highly likely that VR simulation-based emergency skills training can lead to competence in a simulated setting. Although competence in a simulated setting is not necessarily the overall goal of any given medical course, curriculum, or educational activity, the paradigm shift towards competency assessment is reflected here.^66^ It seems that performance assessment in simulation may offer the effectiveness in measuring efficacy of the educational tool, whilst not requiring the resources to competence in the real-world clinical setting.

Alongside the breadth of the included technology-based interventions, we can clearly see the range and complexity of the research outcome measures and the instruments used to assess these. As the interest and evidence strengthens over time, we recommend that the scientific community attempt to form consensus around optimising educational research outcomes, in order to be effective and efficient when validating novel VR educational tools. In the context VR education, we believe that the above consensus is the first step towards development of a framework for evaluation. A single framework designed to enable rapid evaluation across disciplines, agnostic of specific hardware and software deployments, has the potential to encourage the evidence base to grow alongside the rapidly evolving XRT ecosystem.

## Strengths and limitations

This study presents the broadest systematic literature review of simulation-based emergency medical VR training to-date. The search strategy used was broad and comprehensive, but was limited by the inclusion of only English articles. The abstract screening and data extraction processes was performed by two independent, blinded reviewers in order to maximise inter-rater reliability. Seen throughout the review is a broad range of interventions, with widely variable combinations of hardware and software, leading to wide variation in the fidelity of the learning experience. Combined with differences in study design, the data are heterogenous, limiting the extent of numerical analysis such as pooling of data to perform meta-analyses. Finally, due to restrictions on researcher resource, authors were not contacted about missing data which may have provided further clarity in bias assessment.

## Future research

It is seen that VR technology is on a rapid development trajectory, with devices becoming smaller, more powerful, and increasingly ergonomic.^17^, ^77^ Increased knowledge and experience is vital if uptake of immersive technology grows as predicted in the Topol Review (>80% affected by the technology by 2040).^77^ Future work is required to ensure that accredited learning programs (which exist in other emerging technologies such as artificial intelligence and genomics) are designed around XRT. Secondly, if healthcare staff are to benefit from VR as a modality and potentially improve upon the limitations of traditional education, the educational and scientific communities are required to formulate consensus around how best to validate VR education prior to curricula roll-out. Without consensus, and a clear framework for evaluation, high degrees of heterogeneity in the data will remain. Ultimately, this may slow the progress toward the development of evidence-based educational tools capable of educating healthcare workers to deliver high-quality patient care.

## Conclusion

Despite the wide variation in study design and quality, outcomes and associated OMIs, it is clear that there may be educational benefit to using VR in the context of simulation-based emergency skills training. Key benefits may include knowledge gain and retention, skill performance in simulation, acceptability, usability, and validity. Currently, there is insufficient evidence to demonstrate clear cost-effectiveness, or direct improvement of patient, or institutional outcomes at this stage.

Declarations of interest

Dr Tony Payton declares his position as the co-founder of VREvo LTD. Mr Jonathan Abbas is the founder of ExR Solutions Ltd, shareholder of VREvo LTD and has received research grants from ENT-UK and The Royal Society of Medicine in order to support his work.

## CRediT authorship contribution statement

**Jonathan R. Abbas:** Conceptualization, Methodology, Formal analysis, Investigation, Data curation, Writing – original draft, Writing – review & editing, Visualization, Project administration. **Michael M.H. Chu:** Conceptualization, Methodology, Investigation, Writing – review & editing. **Ceyon Jeyarajah:** Formal analysis, Investigation, Data curation, Writing – original draft, Writing – review & editing. **Rachel Isba:** Conceptualization, Methodology, Writing – review & editing, Supervision. **Antony Payton:** Conceptualization, Methodology, Writing – review & editing, Supervision. **Brendan McGrath:** Writing – review & editing, Supervision. **Neil Tolley:** Methodology, Writing – review & editing, Supervision. **Iain Bruce:** Conceptualization, Methodology, Writing – review & editing, Supervision.

## Declaration of Competing Interest

The authors declare the following financial interests/personal relationships which may be considered as potential competing interests: Jonathan Abbas reports financial support was provided by The Royal Society of Medicine. Jonathan Abbas reports financial support was provided by ENT-UK. Jonathan Abbas reports a relationship with ExR Solutions Ltd that includes: board membership, employment, and equity or stocks. Jonathan Abbas reports a relationship with VREvo Ltd that includes: equity or stocks and travel reimbursement. Antony Payton reports a relationship with VREvo Ltd that includes: board membership, employment, and equity or stocks.
