# Supplementary material for: Virtual reality in simulation-based emergency skills training: A systematic review with a narrative synthesis
Source: Resusc Plus. 2023 Oct 21;16:100484. doi: 10.1016/j.resplu.2023.100484 (PMC10618508; doi:10.1016/j.resplu.2023.100484)
Supplement: Supplementary Data 1 [file mmc1.docx]

Appendix 1

**Summary of the key study characteristics of the 34 included articles in this systematic review**

| Title | Citation (publication year) *country* | Study Methodology (Subtype) | Participants - healthcare provider group (total number), further description | Summary of intervention  Technology used to deliver intervention Timing  (number of participants in intervention arm) | Summary of Control  Technology used to deliver intervention Timing  (number of participants in intervention arm) | Outcomes (outcome measure instruments) | Results |
| --- | --- | --- | --- | --- | --- | --- | --- |
| Development and Considerations for Virtual Reality Simulations for Resuscitation Training and Stress Inoculation [28] | Chang TP (2021) *USA* | Randomised controlled trial (Crossover) | Doctors (n=34). Including expert emergency paediatric physicians and paediatric residents. | Paediatric seizure case-based simulation   Oculus Rift HMD and controllers  Single 1 hour session   (n=34) | Paediatric anaphylaxis case-based simulation   Oculus Rift HMD and controllers  Single 1 hour session   (n=34) | 1. Skill performance in VR (time to key interventions)  2. Stress (heart rate and salivary cortisol) | 1. Senior physicians faster to key interventions in the anaphylaxis scenario with no difference in the seizure scenario. Seniors were more likely to progress through the seizure algorithm faster than junior trainees.   2. Overall heart rate of participants were within normal limits with junior physicians having a significantly higher heart rate and higher cortisol level. |
| Immersive and Non-Immersive VR Percutaneous Coronary Intervention Simulation for Acute Myocardial Infarction [29] | Perez-Gutierrez B (2020) *Colombia* | Randomised controlled trial (Parallel) | Medical Students (n=10). All students in 4th year of study. | Primary percutaneous coronary intervention simulation (PCI)   Oculus Rift HMD and controllers  Timing not detailed   (n=5) | Primary percutaneous coronary intervention simulation (PCI)   Android based smartphone application  Timing not detailed   (n=5) | 1. Usability (System Usability Questionnaire (SUS)) | 1. Intervention SUS was 84% compared with 82% for control. No statistical significance calculations were performed. |
| Training on a virtual reality cricothyroidotomy simulator improves skills and transfers to a simulated procedure [58] | Sankaranarayanan G (2022) *USA* | Randomised controlled trial (Parallel) | Medical students (n=20). Second and third year medical students. | Skills trainer to teach surgical cricothyroidotomy  High-fidelity haptic controller and computer screen called Virtual Airway Skills Trainer - Cricothyrotomy (VAST-CCT)  One session a day for 10 consecutive days after watching an expert perform this procedure on the same trainer. Each session was maximum one hour.   (n=10) | No training  (n=10) | 1. Simulator performance (time to completion) and simulation score  2. Skill transfer (Computed performance score and expert rating assessment) | 1. Intervention group completed the task faster at post-intervention simulation and the retention simulation. The intervention improved the speed of simulation pre- and post-intervention.   During the post-test and later the retention tests the intervention group outperformed the control group in terms of simulation score.   2. The overall computed performance scores were higher in the simulation group. Although there was high inter-rater agreement, there was no significant difference in expert rating scores. |
| Impact of an Immersive Virtual Reality Curriculum on Medical Students’ Clinical Assessment of Infants With Respiratory Distress [37] | Zackoff M (2020) *USA* | Randomised controlled trial (Parallel) | Medical students (n=168). All students in 3rd year of study. | Identification of an infant with respiratory distress clinical training  Oculus Rift HMD and controllers and traditional teaching methods including lecture and mannequin simulation  A single educational session with 60 minutes of traditional teaching and 30 minutes of VR  (n=78) | Identification of an infant with respiratory distress clinical training  Traditional teaching methods including lecture and mannequin simulation  A single educational session with 60 minutes of traditional teaching  (n=90) | 1. Skill performance (recognition of examination findings, recognition of correct respiratory distress status and escalation)  2. Self-assessment of competency (pre-and post-self-test of competency) | 1. Intervention group was found to perform higher statistically in several domains across all cases including interpretation of mental state assessment, commenting of specific examination findings and interpretation of vital signs.   2. Significant improvement in the self-assessed scores in the intervention group in 81% of the students. |
| Training Effectiveness of a Wide Area Virtual Environment in Medical Simulation [53] | Wier G (2017) *USA* | Comparative prospective cohort study | Expeditionary Medical Support Course Students (n=470). 86 doctors, 126 nurses and 243 'medics'.  No specific seniority reported | Military traumatic injuries field training during an expedition medicine course   Wide Area Virtual Environment (WAVE)  Single session continued until the scenario was completed   (n=235) | Standard outdoor military field training during an expedition medicine course.  Single session continued until the scenario was completed.  (n=235) | 1. Skill performance in simulation (time to completion, competency skill demonstration, adherence to standardised team performance evaluation tool)  2. Participant observations (self-assessed, post-test evaluation around confidence and realism) | 1. Control group outperformed intervention group in all Standardised Team Performance Evaluation Tool criteria.   2. Intervention groups were overall more satisfied, and had more confidence after the intervention than the control. They rated the realism high when compared to previous training. |
| Performance evaluation of AR/VR training technologies for EMS first responders [32] | Koutitas G (2021) *USA* | Randomised controlled trial (Parallel) | Prehospital Emergency Medical Staff (n=30). Specific roles included Emergency Medical Technicians, and Ambulance Bus Medical Staff. | Intervention 1 - Training modules designed to teach about the features of the ambulance buses (AMBUS)  Oculus Rift HMD and controls in addition to traditional lecture  Use of VR as much as the participant wanted, but at least once on the day of the test and at least three times overall  (n=10)  Intervention 2 -Training modules designed to teach about the features of the ambulance buses (AMBUS)  Oculus Rift HMD and controls in addition to traditional lecture  Use of VR as much as the participant wanted, but at least once on the day of the test and at least three times overall   (n=10) | Training modules designed to teach about the features of the ambulance buses (AMBUS)  Traditional training identical to intervention arm then 60 minute tutorial in the real AMBUS  (n=10) | 1. Performance (overall performance metric - equation combining time and error rate), average time on task, average number of errors | 1. VR training showed improvement in all performance domains over AR and traditional training. |
| Comparative study of a simulated incident with multiple victims and immersive virtual reality[31] | Price MF (2018) *Spain* | Comparative cohort study | Nurses (n=67) | Mass casualty simulation skills training  Samsung Gear VR HMD powered by a Samsung Galaxy S6 smartphone + didactic lecture  VR simulation delivered one year after the clinical simulation  (n=32) | Mass casualty simulation skills training  Clinical simulation with live actors as patients.  Singles session delivered at time '0'  (n=35) | 1. Skill performance during simulation (Percentage of victims correctly triaged and performing critical steps)  2. Stress (basal and post-simulation salivary cortisol levels) | 1. No significant difference noted in skill performance  2. Basal markers of stress were not different between groups. Whilst the difference (increase in stress) before and after increased for the control group more than the intervention group the overall post-test scores were higher in the intervention group.  The level of stress and the performance scores were not correlated. |
| Acquisition of Fire Safety Knowledge and Skills With Virtual Reality Simulation [57] | Rossler KL (2018) *USA* | Randomised controlled trial (Parallel) | Nursing students (n=26) | Educational session design to teach fire safety skills including guideline introduction, operating room role allocation and clinical scenario training  VEST VR trainer (hardware description not provided) and didactic lecture  Traditional lecture based education followed immediately by a VR trainer orientation and finally five independent VR module activities on the VEST trainer  (=13) | Educational session design to teach fire safety skills including guideline introduction, operating room role allocation and clinical scenario training  Traditional didactic lecture  Further detail of timing not given  (n=13) | 1. Knowledge acquisition (knowledge test)  2. Performance (assessed during the course as pass and fail for fire safety training) | 1. Whilst baselines scores were not different between groups intervention groups showed a higher increase in knowledge gained than control.   2. All participants passed therefore no change noted. |
| Decontamination Training With and Without Virtual Reality Simulation [55] | Farra SL (2015) *USA* | Randomised controlled trial (Parallel) | Nursing students (n=106). Senior nursing students from two different undergraduate nursing degrees. | Disaster decontamination skills based learning  A computer and monitor connected to a Microsoft Kinect camera   Single 10 minute VR experience  (n=52) | Disaster decontamination skills based learning  Written instructional sheet describing the process of decontamination  Single 10 minute review of instructions  (n=54) | 1. Skill performance in simulation (time to task completion, assessor rubric score, psychomotor performance checklist)  2. Knowledge acquisition (Multiple Choice Questionnaire adapted from a validated Federal Emergency Management Agency (FEMA) resource)  3. Emergency preparedness (validated Emergency Preparedness Information Questionnaire - EPIQ) | 1. Time to task completion and psychomotor performance scores were improved in the intervention group. Rubric data was missing.   2. No difference in increase in knowledge between groups.   3. Overall EPIQ scores were unchanged between groups however decontamination specific questions showed improvement in self efficacy in the intervention group. |
| Virtual reality triage training provides a viable solution for disaster-preparedness [34] | Andreatta PB (2010) *USA* | Randomised controlled trial (Parallel) | Doctors (n=15). Emergency medicine residents. | Disaster preparedness training  Cave Automatic Virtual Environment (CAVE) plus 1 hour didactic lecture  Single CAVE session immediately after lecture  (n=7) | Disaster preparedness training  Live high fidelity simulation trainer plus 1 hour didactic lecture  Single high-fidelity simulation immediately after the lecture  (n=8) | 1. Skill performance in simulation (32-item triage assessment instrument, total correctly triaged patients)  2. Knowledge acquisition (pre- and post-test questionnaire) | 1. Small effect in favour of intervention for both performance measures.  2. Large effect in favour of the control for knowledge test improvement. |
| MediSim: A prototype VR system for training medical first responder [38] | Stansfield S (1998) *USA* | Randomised controlled trial (Parallel) | Paramedics, Nurses and doctors (n=20). Paramedical staff were specifically emergency medicine technicians and doctors were emergency medicine physicians. One third of the participants had military experience. | Multiple casualty triage in battlefield setting  HMD display and body trackers (brands not given due to prototype design being tested)  Orientation / systems pre-brief followed by a practice-run of the scenario  (n=10) | Multiple casualty triage in battlefield setting  HMD display and body trackers (brands not given due to prototype design being tested)  Orientation / systems pre-brief without an opportunity to practice  (n=10) | 1. Usability (human-system interface assessment)  2. System acceptability (questionnaire) | 1. Intervention group without prior experience of VR had higher task workload.   2. Both groups rated high usability in terms of augmentation to existing rehearsal, and presentation style and ergonomics. Participants preferred higher resolution displays and HMDs were not acceptable due to low field-of-view. |
| 360 virtual reality paediatric mass casualty incident: A cross sectional observational study of triage and out-of-hospital intervention accuracy at a national conference [33] | Lowe J (2020) *USA* | Observational | Doctors and medical students (n=207). Emergency medicine attendings, residents and medical students rotating into the emergency department. | Mass casualty triage  Oculus Go or Daydream VR HMDs and hand controls  Training prior to the assessment was allowed as much as the participant felt necessary. The assessment was immediately after the training.   (n=207) | Not applicable | 1. User experience (Likert questionnaire) | 1. The VR experience was highly engaging and enjoyable, preparing participants for adolescent MCI. Majority felt that VR should be integral to medical education and would like to see more in disaster and paediatric training. Majority of participants felt it was more immersive than simulation skills training. |
| Using Immersive Simulation for Training First Responders for Mass Casualty Incidents [52] | Wilkerson W (2008) *USA* | Observational | Paramedics (n=15). Included paramedics with at least 4 years of experience. | Simulated terrorist attack in sports field training   CAVE   Single 20 minute session immediately after equipment orientation  (n=15) | Not applicable | 1. Performance in simulation (observational assessment)  2. Usability and realism (post-intervention interview) | 1. Majority of participants counted the number of victims but didn’t communicate this to dispatch. A single participant performed a specific safety survey, with a minority alerting hospitals. Five participant identified nature of the incident to the dispatcher but 0 noticed a second explosive device. Performed was therefore mixed by participants.   2. A 10-15 minute orientation was sufficient, and participants took less than 5 minutes to be comfortable with the technology. Repeatability was attractive and there was high levels of immersion and realism. |
| Is individual practice in an immersive and interactive virtual reality application non-inferior to practicing with traditional equipment in learning systematic clinical observation? A randomized controlled trial [30] | Berg H (2020) *Norway* | Randomised controlled trial (Parallel) | Medical and nursing students (n=289). Included only first-year students. | Training around the ABCDE assessment approach  Oculus Rift or Oculus Quest VR HMDs and controllers  Timing as follows: 15 minute introduction, 20 minute individual practice and 15 minute testing period  (n=149) | Training around the ABCDE assessment approach  Individual self-practice on traditional mannequin equipment with written instruction  Timing as follows: 15 minute introduction, 20 minute individual practice 15 minute testing period  (n=149) | 1. Performance (number of students who documented ABCDE correctly and variations of this)  2. Usability (Likert scale questionnaire and SUS) | 1. Non-inferiority between intervention and control  2. Students preferred VR over traditional education with belief that VR is a better way to learn ABCDE approach. SUS scores were higher in the intervention group. |
| Virtual Reality Triage Training Can Provide Comparable Simulation Efficacy For Paramedicine Students Compared To Live Simulation-Based Scenarios [34] | Mills B (2020) *Australia* | Randomised controlled trial (Crossover) | Paramedic students (n=29). Included only students in their third year. | Mass casualty triage simulation skills training  Oculus Rift or HTC Vive VR HMDs and hand associated controls  One-day course with live and VR training done on the same day  (n=29) | Mass casualty triage simulation skills training  Live high-fidelity simulation  One-day course with live and VR training done on the same day  (n=29) | 1. Performance (observational assessment by human or intervention directly and time in simulation)  2. Stress (heart rate)  3. User experience (simulation design scale and focus group discussions)  4. Cognitive load (NASA Task Load Index) | 1. Participants in the control group spent longer in simulation with no difference in score.  2. Stress was higher in live simulation.  3. No significance in user satisfaction. VR is considered a steppingstone into live simulation and cannot replicate emotional realism. VR is graphically realistic, and a higher number believed that the information, support, feedback and fidelity in the intervention was higher than in the control. |
| Virtual reality operating room with AI guidance: design and validation of a fire scenario [35] | Qi D (2021) *USA* | Observational | Doctors and others not specified (n=53). Doctors worked predominantly in emergency medicine. | Operating room fire simulation training  HTC VIVE VR HMD and controls  Following verbal orientation of the task the participants performed 3-5 run-throughs of the VR simulation  (n=53) | Not applicable | 1. Performance (pass rate)  2. User experience (face-validity assessment with Likert scale questionnaire) | 1. Subsequent trials of VR training improved performance in the test scenario. AI guidance improved performance significantly.   2. Majority of participants preferred to learn in VR over traditional methods and agreed it was realistic. Overall usability rating was high. |
| Surgeons With Five or More Actual Cricothyrotomies Perform Significantly Better on a Virtual Reality Simulator [56] | Qi D (2020) *USA* | Observational | Doctors, medical students and undergraduate students (n=47). Doctors vary clinically from student to senior attending. | Cricothyroidotomy skills training  High-fidelity haptic controller and computer screen called Virtual Airway Skills Trainer - Cricothyrotomy (VAST-CCT)   Three practice sessions followed by one assessment session immediately following  (n=47) | Not applicable | 1. Usability (face-validity assessment with Likert scale questionnaire) | 1. Users rated the simulator as highly useful for learning with 8/9 domains being rated as acceptable. The lowest rating corresponded to instrument handling realism. |
| Design and implementation of a virtual reality system and its application to training medical first responders [36] | Stansfield S (2000) *USA* | Observational | Paramedics (n=23). Paramedics of 'extensive' field experience in emergency medicine. | Medical scenario simulation training using pneumothorax or head injury cases.   HMD based VR and hand controls  Introductory video based orientation followed by a 10-minute VR session  (n=23) | Not applicable | 1. System acceptability (18-question system acceptability assessment) | 1. High satisfaction in areas of audio-visual feedback and vital sign indicators. Scenarios were low levels of difficulty and the users denoted moderate satisfaction with field of view and the ability to manipulate objects. |
| Virtual reality vs. high-fidelity manikin-based simulation: A randomized comparison trial on case leadership skills [39] | Abulfaraj MM (2021) *USA* | Randomised controlled trial (Crossover) | Doctors (n=42). Including paediatric and emergency medicine interns | Medical scenario simulation training using status epilepticus and anaphylaxis scenarios  Oculus Rift HMD and controls  After 5-minute orientation, two 30 minute VR sessions were undertook  (n=22) | Medical scenario simulation training using status epilepticus and anaphylaxis scenarios  Live high-fidelity simulation.   After 5-minute orientation, two 30 minute simulation sessions were undertook  (n=20) | 1. Performance (time to complete critical actions)   2. Confidence (self-reported confidence questionnaire)  3. System Usability (SUS) | 1. No significant difference noted in skill performance.  2. No significant difference in reported confidence.   3. High usability scores were observed. |
| Virtual reality enhanced mannequin (VREM) that is well received by resuscitation experts [47] | Semeraro F (2009) *Italy* | Observational | Doctors, nurses and lay responders (n=39). | Cardiopulmonary resuscitation (CPR) skills training  VR HMD and mannequin combined  Single session timing not detailed  (n=39) | Not applicable | 1. Usability (Likert scale with the domains of realism, immersion and user-friendliness) | 1. High levels of realism and immersion noted. The majority of participants had no difficulty wearing the device however eight found it difficult with some participants finding it difficult to reach the patient. Majority believed it to be potentially useful educationally. |
| Effects of Virtual Reality Simulation on Worker Emergency Evacuation of Neonates [61] | Farra S (2019) *USA* | Randomised controlled trial (Unclear) | Doctors, Nurses, Monitor technicians and Respiratory physiotherapists (n=94). | Worker emergency evacuation of neonates training  VR HMD with keyboard and mouse inputs. If discomfort then switched to screen based equivalent  Single session repeated at 0, 4, 8 and 12 months  (n=unclear from full-text)   All users underwent web-based modules also. | Worker emergency evacuation of neonates training  Computer based, non-immersive scenarios using the VR storyboard images and a question / answer software interface  Single session repeated at 0, 4, 8 and 12 months  (n=unclear from full-text) | 1. Knowledge (cognitive assessment questionnaire)  2. Performance (validated psychomotor skills performance assessment).   3. Emergency preparedness (EPIQ questionnaire) | 1. Knowledge improved overall between groups but there was no difference between control and intervention groups.   2. Intervention group performed better overall.   3. Preparedness improved overall and in neonatal specific components. |
| Teaching mass casualty triage skills using immersive three-dimensional virtual reality [48] | Vincent D (2008) *USA* | Observational | Medical students (n=24). All years of study were included with 60% in the first year. | Mass casualty triage training  Fifth Dimension Technologies HMD, stereo headphones and 3 motion tracking Ascension Technology sensors  Three sessions one after another  (n=24) | Not applicable | 1. Performance (Triage score, intervention score, time to triage)  2. Learner satisfaction (self-assessed questionnaire)  3. Self-rated efficacy (self-assessed questionnaire) | 1. Scores improved between intervention rounds A and B. Time to completion continued to improve across all three intervention rounds.   2. Satisfaction was high including belief that the course was presented at the appropriate level, was not too fast in pace, and was relevant.   3. Confidence improved specifically relating to belief that students have the ability to be first responders, and improving one’s confidence to assess mass casualty events. |
| Resuscitating Cardiopulmonary Resuscitation Training in a Virtual Reality: Prospective Interventional Study [45] | Perron JE (2021) *Australia* | Observational | Medical students (n=26). | Virtual Doc CPR training.   Oculus Rift HMD and hand controllers  1 hour VR session  (n=26) | Not applicable | 1. Participant satisfaction (self-assessed questionnaire with domains around educational effectiveness, overall satisfaction and gamification) | 1. Majority of participants enjoyed the simulation and would recommend it to a colleague or friend. The majority also perceived it to be educationally valuable in terms of preparing for real scenario and increased understanding of CPR. Participants agreed that gamification enhanced the experience and potential learning. |
| Use of Virtual Reality for Paediatric Cardiac Critical Care Simulation [46] | Ralston BH (2021) *USA* | Observational | Doctors (n=6). All participants were attending paediatric critical care physicians. | Paediatric cardiac critical care simulation training  Oculus Quest headset and controllers  Two VR simulations delivered sequentially with one run-through of each  (n=6) | Not applicable | 1. Participant satisfaction (self-assessed questionnaire) | 1. Unanimous agreement that the scenarios were true to life and enjoyable. The VR environment was felt to be realistic in a close majority with 83.3% believing VR enhanced the simulation experience. |
| Comparing the Effects on Learning Outcomes of Tablet-Based and Virtual Reality-Based Serious Gaming Modules for Basic Life Support Training: Randomized Trial [40] | Aksoy E (2019) *Turkey* | Randomised controlled trial (Parallel) | Paramedic students (n=40). All included were in their first year of study. | Basic Life Support (BLS) training session   VR HMD (brand not specified)  One session in VR immediately after an orientation session  (n=22) | Basic Life Support (BLS) training session   Tablet based serios game   One session on the serios game immediately after an orientation session  (n=18) | 1. Knowledge (knowledge test) | 1. Both the intervention and control improved knowledge with the greatest improvement in intervention group. |
| Design of a Serious Game for Handling Obstetrical Emergencies [43] | Gautier JD (2016) *France* | Observational | Doctors and student midwives (n=not specified). | Obstetric emergency simulation skills training  Oculus Rift and controllers   Four simulations delivered with timings unclear  (n=unclear) | Not applicable | No evidence of any primary outcome measures  Players provided with a score table of 5 sections based on their performance.  These scores not collated as part of paper. | Not applicable |
| An Immersive Multi-User Virtual Reality for Emergency Simulation Training: Usability Study [44] | Lerner D (2020) *Germany* | Observational | Doctors (n=18). Emergency physicians. | Paediatric anaphylaxis with grade III shock  HTC Vive Pro HMD and controller with PCs for controlling the simulation  Following briefing, and VR familiarisation. 20 mins VR training scenario.  (n=18) | Not applicable | 1. Training effectiveness (Training Evaluation Inventory (TEI)) Measurement of additional variables regarding media use.   2. Levels of presence (group presence questionnaire (IPQ))  3. Usability (SUS)  4. Knowledge (knowledge test score)  5. Motivation (simulation motivation score (SIMS)) | 1. High levels of training effectiveness observed.  2. High levels of presence correlated significantly with perceived training effectiveness.   3. Usability was rated high and was significantly correlated with IPQ scores. |
| Virtual reality mobile application to improve videoscopic airway training: A randomised trial [59] | Yau YW (2021) *Singapore* | Randomised controlled trial (Parallel) | Doctors (n=45). Doctors of all grades from the emergency department. | Airway skills training.   Airway Ex mobile application with VR hardware which is unspecified  Thirty minutes self-directed learning using the mobile app (Airway Ex) across a total of six cases  (n=22) | Airway skills training.   Conventional didactic teaching and low fidelity simulation using a mannequin.   Thirty minutes self-directed learning using the mobile app (Airway Ex) across a total of six cases  (n=23) | 1. Performance (time to key milestones, proportion of successful intubations and quality of scope manipulation)  2. Confidence (self-reported change confidence score) | 1. No significant difference in time to critical milestones, however 2 control group participants failed the activity. Manipulation of the scope was higher in quality in the intervention group when adjusted also for age, gender, seniority and prior experience.   2. No differences seen in confidence. |
| The Impact of Virtual Reality Simulation on Cognitive Achievement of Nursing Students [60] | Permana RH (2019) *Indonesia* | Randomised controlled trial (Parallel) | Nursing students (n=27). | Intervention one: Cognitive training in respiratory distress in the context of nursing problems, interventions and theoretical knowledge   Lecture and VR training. Hardware not specified however it was built upon android architecture   Sixty minute lecture followed by 17 minute VR session  (n=9)  Intervention two: Learning outcomes were identical  VR training only with the same application as in intervention one   17 minute VR session only  (n=9) | Cognitive training in respiratory distress in the context of nursing problems, interventions and theoretical knowledge  Didactic lecture (participants were given opportunity to us VR after the testing period had concluded therefore not contributing to the data)  Sixty minute single lecture  (n=9) | 1. Knowledge (knowledge test score) | 1. A significant difference was seen between groups. Knowledge test scores were highest in VR only group and lowest in VR + lecture combined. |
| Simulator Training Improves Fibre-optic Intubation Proficiency among Emergency Medicine Residents [54] | Binstadt E (2008) *USA* | Observational | Doctors (n=21). Emergency medicine residents. | Fibreoptic intubation skills training  AccuTouch endoscopy simulator (non-HMD VR)  Participants performed three simulated bronchoscopy cases in same order. Subsequent 20 minute VR tutorial and further 10 minute practice and finally repeating the three simulated bronchoscopy cases repeated in the same order.  (n=21) | Not applicable | 1. Skill performance (time to intubation, endoscope collisions and efficiency score) | 1. Performance increased in all domains from initial to repeat simulations. |
| OR fire virtual training simulator: design and face validity [42] | Dorozhkin D (2017) *USA* | Observational | Doctors, medical students (n=49). Any attendee to the conference, but predominantly surgical background or interest. | Operating theatre fire training  Oculus Rift HMD and a single handheld interaction device.   Timing of intervention not clear  (n=49) | Not applicable | 1. Perceived educational effectiveness (Likert scale self-assessed questionnaire) | 1. Majority of domains were rated highly. The lowest score was around haptic feedback and feeling of the devices. Overall usefulness was very high and majority participants would choose VR over traditional training. |
| A virtual reality methodology for cardiopulmonary resuscitation training with and without a physical mannequin [41] | Buttussi F (2020) *Italy* | Randomised controlled trial | Undergraduate non-medical students (n=30). Computer science undergraduates. | CPR training  HTC Vive Pro HMD with two Lighthouse base stations and two HTC trackers for the wrists. The mannequin - Laerdal Resusci Anne, with two additional HTC trackers to synchronise its position with VR. With two sensors on the Arduino board inside the mannequin.  Two trials of CPR were performed with 30 compressions in each trial performed twice  (n=15) | CPR training  HTC Vive Pro HMD with two Lighthouse base stations and two HTC trackers for the wrists   Two trials of CPR were performed with 30 compressions in each trial performed twice  (n=15) | 1. Performance during simulation training (markers of high and low quality CPR collected by software)  2. Performance during simulation assessment (markers of high and low quality CPR collected by software, and knowledge of the algorithm)  3. Self-efficacy (self-assessed questionnaire) | 1. Results were mixed with some markers of CPR effectiveness favouring the intervention and some favouring control.   2. Results were mixed with some markers of CPR effectiveness favouring the intervention and some favouring control. In terms of algorithmic knowledge statistically significant difference was seen only in one domain (number of wrong steps) which favoured the control group.  3. Self-efficacy improved for both groups but no difference between groups was seen. |
| The Use of Immersive and Virtual Reality Technologies to Enable Nursing Students to Experience Scenario-Based, Basic Life Support Training-Exploring the Impact on Confidence and Skills [51] | Rushton MA (2020) *UK* | Comparative cohort study | Nursing students (n=208). Undergraduate students in the 2nd year of study. | BLS simulation skills training  Octave CAVE VR with Laerdal QCPR manikin  Single simulation performed.  (n=80) | BLS simulation skills training  Non-immersive Laerdel mannequin CPR training  Single simulation performed.  (n=55) | 1. Skill performance (mannequin scores including overall BLS score, compression score, ventilation score, and data regarding compressions and ventilations)  2. Learner satisfaction (Likert scale questionnaire) | 1. Mean overall scores were lower in the intervention group than the control group. When interrogating individual scores some favoured the intervention and some the control.  2. The intervention was described as giving insight into real situation, compared to the control being a relaxed educational environment. Intervention equipment was rated higher than the control. The intervention was felt to be a very good learning tool which prepares participants for real situations. |
| Development and evaluation of a trauma decision-making simulator in Oculus virtual reality [49] | Harrington CM (2018) *Ireland* | Observational | Doctors (n=26). Course instructors were expert level doctors of varying specialties and course candidates were novice doctors. | Advanced Trauma Life Support (ATLS) style moulage training case on the RCSI medical training simulator package (https://www.oculus.com/experiences/gear-vr/878262692296965)  Samsung Gear VR HMD powered by a Samsung Galaxy S6 or S7-Edge device  Orientation to technology video followed by single use of the application | Not applicable | 1. Performance during simulation (number of correct decisions, number of incorrect decisions, number of correct diagnoses, number of incorrect diagnoses, number of patient deaths and time taken)  2. Participant satisfaction (Likert scale questionnaire)  3. Knowledge – ATLS course MCQs | 1. Correlations were found between high candidate knowledge and high simulator performance. Instructors scored significantly higher than course participants.   2. Feedback related to hardware was mostly positive. Moderate / high evaluation scores were given for immersion, realism and appearance of simulation and cost-effectiveness. It was rated as highly enjoyable. The simulator was regarded as a useful teaching tool, but criticism was presented due to the lack of content. |
